# Activating mutations in ALK kinase domain confer resistance to structurally unrelated ALK inhibitors in NPM-ALK-positive anaplastic large-cell lymphoma

**DOI:** 10.1007/s00432-014-1589-3

**Published:** 2014-02-08

**Authors:** Daria Zdzalik, Barbara Dymek, Paulina Grygielewicz, Pawel Gunerka, Anna Bujak, Monika Lamparska-Przybysz, Maciej Wieczorek, Karolina Dzwonek

**Affiliations:** 1 Innovative Drugs R&D Department, Celon Pharma Inc., Mokra 41a, 05-092 Lomianki/Kielpin, Poland; 2 Department of Immunology, Center for Biostructure Research, Medical University of Warsaw, Banacha 1a, F Building, 02-097 Warsaw, Poland

**Keywords:** ALK, ALCL, Drug resistance, ALK inhibitors, Crizotinib, CH5424802

## Abstract

**Purpose:**

Crizotinib, the first FDA-approved ALK inhibitor, showed significant antitumor activity in young patients with anaplastic large-cell lymphoma (ALCL) frequently displaying *ALK* rearrangement. However, long-term therapeutic benefits of crizotinib are limited due to development of drug resistance. CH5424802—more potent and selective ALK inhibitor—comprises a good candidate for second-line treatment in crizotinib-relapsed patients. The aim of this study was to determine possible mechanisms of resistance to ALK inhibitors that can appear in ALCL patients.

**Methods:**

ALK+ ALCL cell lines resistant to crizotinib (Karpas299CR) and to CH5424802 (Karpas299CHR) were established by long-term exposure of Karpas299 cells to these inhibitors. Next, alterations in their sensitivity to ALK, HSP90 and mTOR inhibitors were investigated by cell viability and BrdU incorporation assays and immunoblot analysis.

**Results:**

cDNA sequencing of *ALK* kinase domain revealed activating mutations—I1171T in Karpas299CR and F1174C in Karpas299CHR. The resistant cells displayed diminished sensitivity to structurally unrelated ALK inhibitors—crizotinib, CH5424802 and TAE684. Nevertheless, CH5424802 and TAE684 were still more potent against the resistant cells than crizotinib. Moreover, Karpas299CR and Karpas299CHR cells remained sensitive to HSP90 or mTOR inhibitors.

**Conclusions:**

Resistance mediated by activating mutations in ALK kinase domain may emerge in ALCL patients during ALK inhibitors treatment. However, more potent second-generation ALK inhibitors, HSP90 or mTOR inhibitors may represent an effective therapy for relapsed ALK+ ALCL patients.

**Electronic supplementary material:**

The online version of this article (doi:10.1007/s00432-014-1589-3) contains supplementary material, which is available to authorized users.

## Introduction

Anaplastic lymphoma kinase (ALK) was initially discovered in anaplastic large-cell lymphoma (ALCL) as a component of fusion protein NPM-ALK formed as a result of the t(2;5)(p23;q35) chromosomal translocation (Morris et al. 1994; Shiota et al. 1995). The discovery of EML4-ALK fusion in 2007 as oncogenic driver in non-small-cell lung cancer (NSCLC) and next the identification of activating mutations in *ALK* gene in neuroblastoma made ALK one of the most extensively studied targets in the field of kinase inhibitor drug development (Chen et al. 2008; George et al. 2008; Janoueix-Lerosey et al. 2008; Mosse et al. 2008; Soda et al. 2007). Until now, the essential role of different ALK fusion proteins has been demonstrated in several neoplasms, such as diffuse large-B-cell lymphoma, inflammatory myofibroblastic tumor, squamous cell carcinoma of the esophagus and renal cell carcinoma (Kruczynski et al. 2012; Palmer et al. 2009). The ALK fusion partner induces homodimerization leading to constitutive ALK kinase domain (KD) activation (Bischof et al. 1997). Aberrant ALK activation triggers prosurvival signaling pathways such as JAK/STAT3, PI3K/AKT and MAPK/ERK pathways (Bai et al. 2000; Chiarle et al. 2005; Marzec et al. 2007b; Palmer et al. 2009) and in consequence drives oncogenesis (Chiarle et al. 2003; Palmer et al. 2009; Soda et al. 2007).

ALK-positive ALCL accounts for 55 % of systemic ALCL, a subtype of T-cell non-Hodgkin lymphoma (Savage et al. 2008; Vose et al. 2008). The most frequent aberration in ALK+ ALCL is the *NPM*-*ALK* fusion (Morris et al. 1994; Swerdlow et al. 2008). Standard treatment for ALCL is based on a high-dose polychemotherapy with autologous stem cell transplantation (Jacobsen 2006). Although the majority of patients respond to the therapy, new treatments are needed for resistant or relapsing patients (Foyil and Bartlett 2012; Schmitz et al. 2010) and there is much hope in ALK inhibitors. There are currently four ongoing clinical trials of crizotinib (NCT00939770, NCT01606878, NCT01524926, NCT00585195) and one of a dual ALK/EGFR inhibitor AP26113 (NCT01449461) in ALCL patients.

Crizotinib, the first dual ALK/MET inhibitor that entered clinical trials, has recently been approved for the treatment of locally advanced or metastatic *ALK*-rearranged NSCLC (Camidge et al. 2012; Christensen et al. 2007). In addition, recently published results of a phase 1 trial in children show that seven out of nine children with recurrent or refractory ALCL experienced a complete response following crizotinib monotherapy (Mosse et al. 2013). This high response rate to crizotinib and its favorable toxicity profile makes it very probable to be approved for ALCL treatment in the nearest future (Mosse et al. 2013). However, based on the data obtained from NSCLC patients treated with crizotinib, it is highly probable that resistance to the therapy would emerge also in ALCL patients, which would limit further clinical benefit of crizotinib-based therapy (Choi et al. 2010; Doebele et al. 2012; Katayama et al. 2011, 2012).

The key mechanism of resistance to crizotinib is secondary mutations within ALK KD, including F1174L, C1156Y, G1202R, S1206Y, G1269A and the gatekeeper mutation L1196M (Choi et al. 2010; Doebele et al. 2012; Katayama et al. 2011, 2012; Sasaki et al. 2010). These resistance mutations can be divided into two main categories. The first one includes mutations of the residues that make the direct contact with inhibitor, thus impairing its binding due to steric hindrance. The second class includes mutations of the residues distant from inhibitor-binding site that promote conformational changes increasing ALK kinase activity (Sasaki et al. 2011; Zhang et al. 2011). Additionally, gain in *ALK* copy number, loss of *ALK* gene rearrangement and activation of alternative signaling mediated by increased phosphorylation of EGFR, amplification of *KIT* or KRAS mutation have also been implicated in the development of acquired resistance to crizotinib (Doebele et al. 2012; Katayama et al. 2012; Sasaki et al. 2011).

The acquired crizotinib resistance mediated by mutations in ALK KD could be overcome by second-generation ALK inhibitors (Katayama et al. 2011, 2012). Promising results were shown for CH5424802, potent and more selective ALK inhibitor with unique scaffold structurally unrelated to crizotinib (Sakamoto et al. 2011). The effectiveness of CH5424802 against L1196M and C1156Y mutations makes it a good candidate for second-line treatment in patients who failed to respond to crizotinib, which is currently studied in clinical trial (NCT01588028) (Sakamoto et al. 2011; Seto et al. 2013).

Since there is lack of information regarding possible mechanisms of resistance to ALK inhibitors that can appear in ALCL patients, we established human NPM-ALK+ ALCL Karpas299 cell line resistant to crizotinib and CH5424802. We found that I1171T and F1174C mutations in ALK KD emerge as a mechanism of acquired resistance to crizotinib and CH5424802, respectively. These mutations resulted in diminished inhibition of ALK signaling and the efficacy of structurally unrelated ALK inhibitors. However, the resistant cell lines still responded to nanomolar concentrations of CH5424802 or TAE684. Moreover, we showed that HSP90 and mTOR inhibitors can be considered as an alternative therapeutic approach in naïve and resistant to ALK inhibitors ALK+ ALCL patients.

## Materials and methods

### Compounds and cell lines

CH5424802 and crizotinib were purchased from Active Biochemicals and Selleck Chemicals, respectively. All remaining inhibitors were provided by LC Laboratories. The human NPM-ALK+ ALCL cell line Karpas299 was purchased from DSMZ and cultured in RPMI 1640 medium supplemented with 10 % fetal bovine serum according to the manufacturer’s instructions.

### Generation of drug-resistant Karpas299 cells

To generate drug resistance, Karpas299 cells were cultured with increasing concentrations of crizotinib or CH5424802, starting from concentrations reflecting 10 % of IC_50_ values (25 and 5 nM, respectively) until reaching the concentration of 2 μM for crizotinib and 100 nM for CH5424802. The resistant cells were cultured until they displayed growth kinetics similar to untreated parental cells.

### cDNA sequencing of coding fragment of ALK kinase domain

Total RNA was isolated from 5 × 10^5^ cells and retrotranscribed, and obtained cDNA was used in PCR with self-designed primers flanking ALK KD coding region: ALK-FW 5′-TCAGTGACCTGAAGGAGGTG-3′ and ALK-REV 5′-AGCCACGTGCAGAAGGTC-3′. The PCR products were sequenced in DNA Sequencing Laboratory, Institute of Biochemistry and Biophysics, Polish Academy of Sciences.

### Cell viability assays

For viability experiments, 1 × 10^4^ cells per well were seeded in 96-well plates and exposed to increasing concentrations of tested inhibitors for 72 h. Cell viability was determined by ATPlite assay (Perkin Elmer) according to the manufacturer’s instructions. IC_50_s and statistical analysis were calculated using GraphPad Prism version 5. The curves were fit using a nonlinear regression model with a log (inhibitor) versus response formula.

### Phosphoprotein array analysis

Cells were incubated in the inhibitor-free medium for 24 h, and then, analysis of phosphorylation status of the panel of key signaling molecules was performed using the PathScan^®^ RTK Signaling Antibody Array (Cell Signaling Technology) according to the manufacturer’s protocol.

### Immunoblot analysis

Cells were seeded in 6-well plates at a density of 0.5 × 10^6^/ml in the inhibitor-free medium. After 24 h, cells were incubated with the indicated concentrations of inhibitors for 2 or 24 h. Next, cells were lysed and examined by Western blotting according to the protocols provided by the antibody suppliers. Primary antibodies against pALK (Y1604), total ALK, pSTAT3 (Y705), total STAT3, pERK1/2 (T202/Y204), total ERK1/2 and pS6 (S240/244) were purchased from Cell Signaling Technology. Antibodies against β-tubulin and GAPDH were obtained from Millipore and Santa Cruz Biotechnology, respectively. Secondary HRP-conjugated antibodies were purchased from Cell Signaling Technology.

### DNA synthesis and cell cycle analysis

Cells (5 × 10^5^/well in 6-well plates) were cultured in the inhibitor-free medium for 24 h and then incubated for 48 h with inhibitors at indicated doses. For replicated DNA staining, incorporated BrdU was probed with anti-BrdU antibody and genomic DNA was stained with 7-AAD following the manufacturer’s procedures (BrdU Flow Kit, BD Pharmingen). Cells were analyzed with FACSCalibur flow cytometer (BD Biosciences) using CellQuest Software.

## Results

### Activating mutations confer resistance to crizotinib and CH5424802 in NPM-ALK+ Karpas299 cells

To establish NPM-ALK+ ALCL Karpas299 cell lines resistant to crizotinib and CH5424802, two structurally unrelated ALK kinase inhibitors, Karpas299 cells were cultured with increasing concentrations of inhibitors for about 4 months. We managed to reach the final concentration of 2 μM for crizotinib and 100 nM for CH5424802. Despite several attempts, we were unable to obtain cell lines resistant to higher concentrations of CH5424802. The established resistant cell lines, designated as Karpas299CR (crizotinib resistant) and Karpas299CHR (CH5424802 resistant), were maintained in final concentrations of inhibitors and were growing with similar kinetics as untreated parental line.

**Fig. 1:**
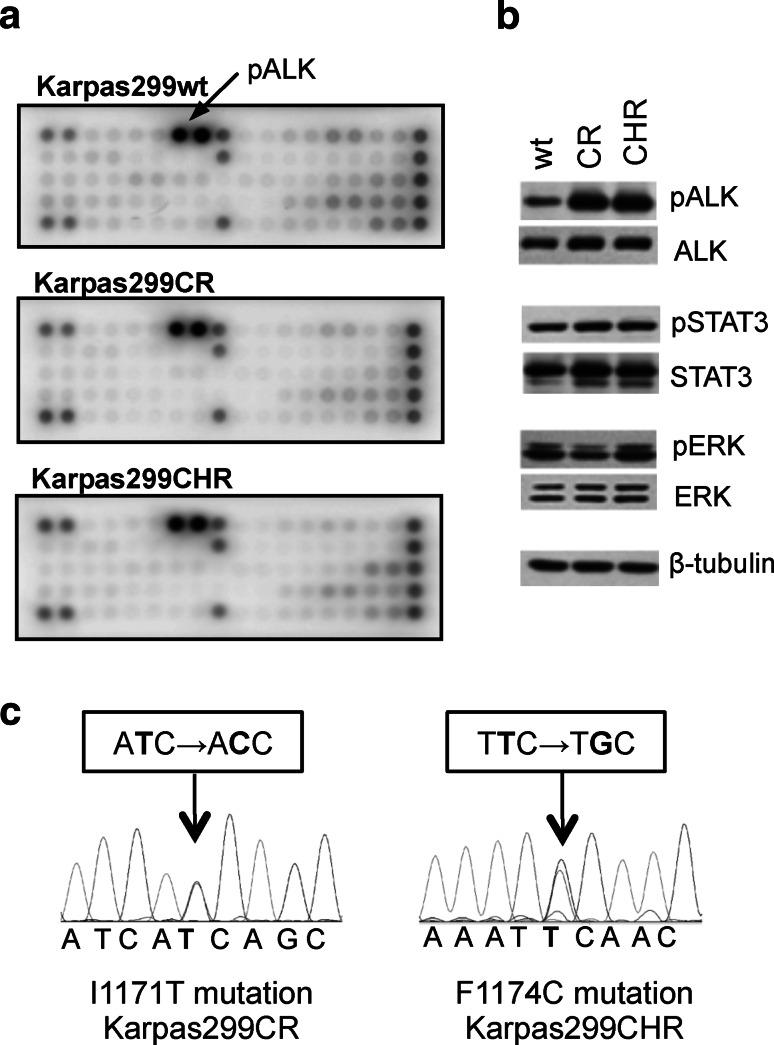
Identification of the resistance mechanism in Karpas299CR and Karpas299CHR cells. **a** Analysis of phosphorylation status of key signaling molecules assessed by the PathScan^®^ RTK Signaling Antibody Array. **b** Immunoblot analysis of pALK and downstream signaling proteins following 24 h of incubation in the inhibitor-free medium. **c** Sequencing of ALK KD coding fragment in Karpas299CR and Karpas299CHR cells

In order to verify whether acquired resistance of Karpas299CR and Karpas299CHR cells is the effect of the induction of alternative signaling pathway, we used the slide-based PathScan^®^RTK Signaling Antibody Array Kit. This analysis revealed that both resistant cell lines sustained high ALK phosphorylation status without activation of any other receptor tyrosine kinases (Fig. 1a). Moreover, both resistant cell lines showed significant increase in ALK phosphorylation status when compared to the parental cells as assayed by immunoblot analysis (Fig. 1b) and measured by densitometry (data not shown). Although pALK levels were significantly higher in Karpas299CR and Karpas299CHR cells, we did not observe corresponding increase in phosphorylation status of downstream proteins such as STAT3 or ERK (Fig. 1b).

Higher levels of ALK phosphorylation suggested that both resistant lines might harbor an activating mutation within ALK KD (Sasaki et al. 2010). Therefore, we analyzed the entire coding sequence of ALK KD in parental and resistant cells. In Karpas299CR cells, we identified a T → C substitution resulting in the change from isoleucine to threonine at position 1171 (I1171T), whereas in Karpas299CHR cells, we detected a T → G substitution that triggers the conversion from phenylalanine to cysteine at position 1174 (F1174C) (Fig. 1c). There were no mutations found in ALK KD sequence of parental cells.

### Karpas299CR and Karpas299CHR cells are less sensitive to crizotinib and CH5424802 compared to parental Karpas299 cells

**Fig. 2:**
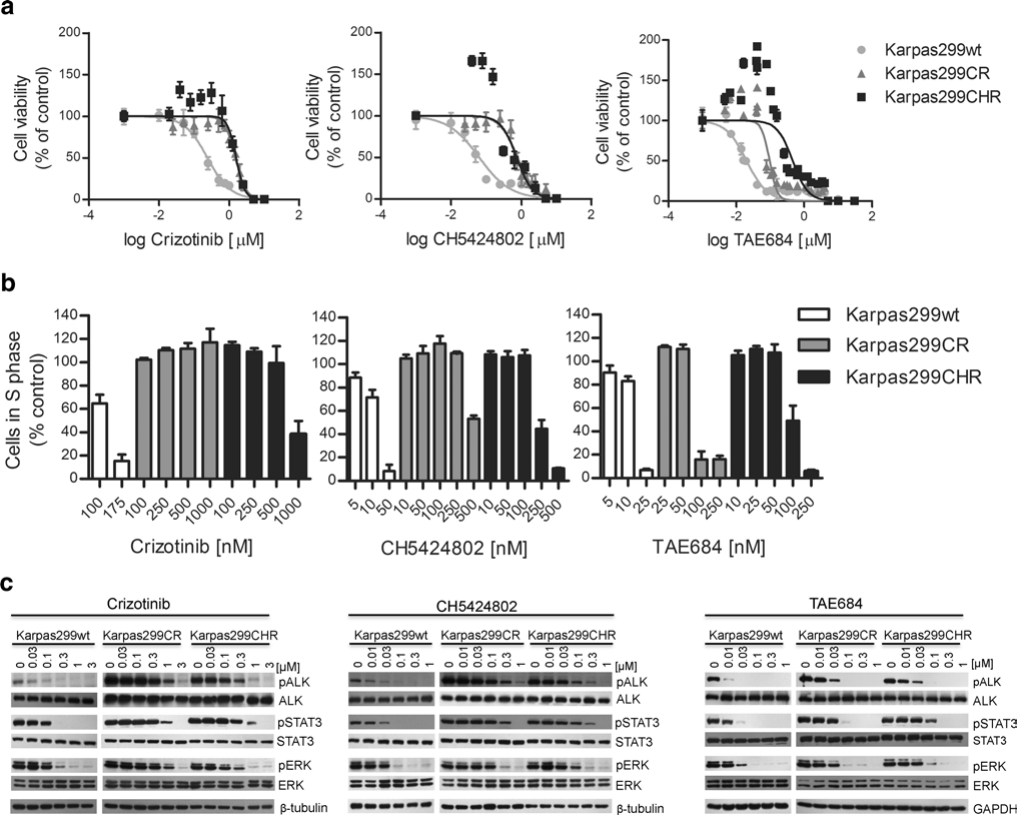
Sensitivity of Karpas299wt and resistant cells to ALK inhibitors. **a** ATPlite cell viability assay following 72 h of incubation with ALK inhibitors. Data expressed as percentage viability relative to untreated controls. Corresponding IC_50_ values are summarized in Table 1. **b** BrdU cell proliferation assay and flow cytometry analysis following 48-h incubation with inhibitors. Data expressed as percentage of cells in S phase relative to untreated control. **c** Immunoblot analysis of pALK and downstream signaling following incubation with inhibitors for 2 h

To assess the susceptibility of newly generated Karpas299CR and Karpas299CHR cells to crizotinib and CH5424802, we tested the influence of these inhibitors on cell growth rate and phosphorylation status of ALK and its downstream effectors. In the cell viability assay, we confirmed that Karpas299CR and Karpas299CHR cell lines were resistant to crizotinib and CH5424802, respectively. In Karpas299CR cells, bearing the I1171T mutation, the IC_50_ value for crizotinib was sixfold higher than in parental cells (*p* < 0.0001). Karpas299CHR cells, bearing the F1174C mutation, responded to CH5424802 with the IC_50_ value almost 12-fold higher than parental cells (*p* < 0.0001) (Table 1). Moreover, Karpas299CR and Karpas299CHR cells exhibited cross-resistance to CH5425802 and crizotinib, respectively (Fig. 2a). In accordance with data obtained from the cell viability assay, crizotinib and CH5424802 treatment had limited effect on proliferation of both generated resistant cell lines in comparison with parental cells. Crizotinib treatment up to 1 μM in Karpas299CR and up to 500 nM in Karpas299CHR did not cause any changes in a number of proliferating cells. CH5424802 doses up to 250 and 100 nM did not reduce proliferation of Karpas299CR and Karpas299CHR cells, respectively (Fig. 2b, Supplementary Fig. S1a and S1b). However, it is worthy of note that in the viability and BrdU incorporation assays, CH5424802 exhibited greater potency in comparison with crizotinib, not only against parental, but also against resistant cell lines suppressing growth and proliferation of cells at nanomolar range. In agreement with the observed resistance, phosphorylation of ALK and its crucial downstream signaling intermediates ERK1/2 and STAT3 was preserved at substantially higher doses of crizotinib or CH5424802 in the resistant cell lines in comparison with wild-type cells (Fig. 2c). Additionally, immunoblot analysis confirmed occurrence of cross-resistance to crizotinib and CH5424802 in the generated resistant cell lines (Fig. 2c).

**Table 1:** IC_50_ values of ALK inhibitors for the growth inhibition of Karpas299 parental (wt), Karpas299CR (CR) and Karpas299CHR (CHR) cell lines obtained by ATPlite assay

Inhibitor	IC_50_ [μM]			Fold change in IC_50_ compared with wt	
	Cells				
	wt	CR	CHR	CR	CHR
Crizotinib	0.239	1.500	1.575	6.3	6.7
CH5424802	0.063	0.771	0.730	12.2	11.6
TAE684	0.020	0.095	0.457	4.8	22.9

The IC_50_ values for the growth inhibition of resistant cells were significantly higher than corresponding values for the parental cells; IC_50_ value for TAE684 against Karpas299CHR was significantly higher than against Karpas299CR (*p* < 0.0001; Akaike information criterion)

### Karpas299CR and Karpas299CHR cells display different level of resistance to the ALK inhibitor TAE684

In order to determine whether the resistant cell lines are sensitive to other, structurally distinct ALK inhibitor, we tested their susceptibility to TAE684—a highly active small-molecule ALK TKI (tyrosine kinase inhibitor) (Galkin et al. 2007). We confirmed that TAE684 exhibited higher potency in cell viability assay in comparison with both crizotinib and CH5424802 (Fig. 2a). TAE684 significantly reduced proliferation of parental Karpas299 cells and to a lesser extent of Karpas299CR, but had modest effect on viability of Karpas299CHR cell line. The IC_50_ values observed with TAE684 were almost fivefold higher (*p* < 0.0001) in the case of Karpas299CR cells and 23-fold higher in the case of Karpas299CHR (*p* < 0.0001) cells as compared with parental cells. Although Karpas299CR cells exhibited some resistance to TAE684, the determined IC_50_ was in low nanomolar range. Moreover, TAE684 treatment of both resistant cell lines markedly reduced the fraction of S phase cells, albeit similar effect on wild-type Karpas299 cells was achieved at much lower doses of this inhibitor (Fig. 2b, Supplementary Fig. S1c). In agreement with determined IC_50_ values, higher concentrations of TAE684 were required to completely suppress proliferation of Karpas299CHR than Karpas299CR cells. As shown in Fig. 2c, exposure of cells to TAE684 markedly inhibited the phosphorylation of ALK and downstream proteins STAT3 and ERK1/2, in a concentration-dependent manner. This effect was reduced in the resistant cell lines in comparison with wild-type Karpas299. Although Karpas299CHR cells exhibit much higher resistance to TAE684 than Karpas299CR cells as revealed by viability and proliferation assays, the effect of the inhibitor on the level of phosphorylated ALK was comparable in both cell lines.

### HSP90 inhibitor AUY922 is highly active against parental and resistant Karpas299 cells

**Fig. 3:**
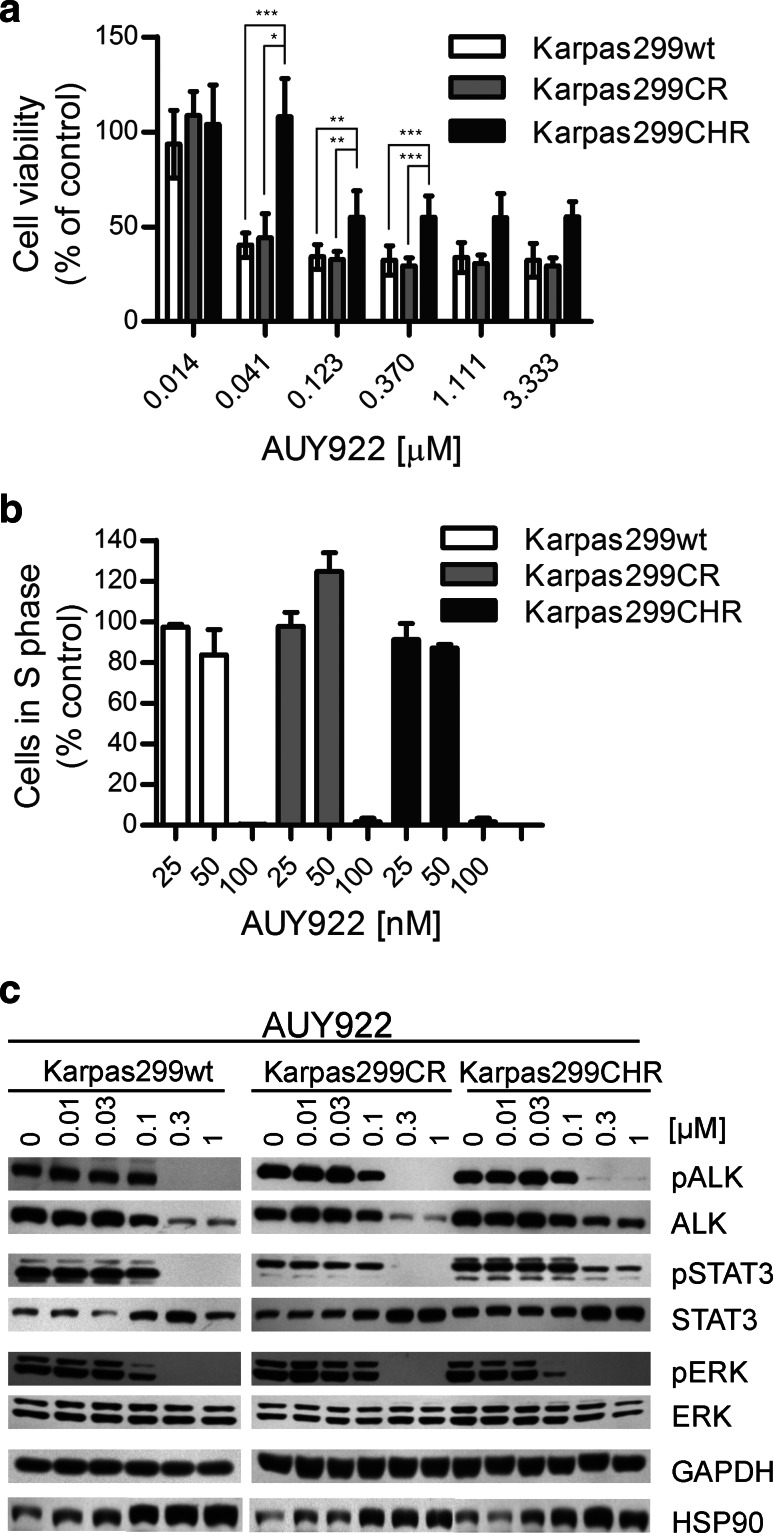
Karpas299CR and Karpas299CHR sensitivity to HSP90 inhibitor AUY922. **a** ATPlite cell viability assay following 72-h incubation with AUY922. Values expressed as percentage viability relative to the untreated controls. *Error bars* represent (±SD) of triplicates obtained from at least three independent experiments. *p ≤ 0.05, **p ≤ 0.01 and ***p ≤ 0.001 (Dunn’s multiple comparison test) for the indicated comparisons. **b** BrdU cell proliferation assay and flow cytometry analysis following 48-h incubation with HSP90 inhibitor. Data expressed as percentage of cells in S phase relative to the untreated controls. Values represent the mean ± SD of two 2 independent experiments. **c** Immunoblot analysis of pALK and downstream signaling following 24-h incubation with AUY922

ALK fusion proteins are HSP90 clients, and it has already been shown in preclinical and clinical studies that HSP90 inhibition is highly effective against EML4-ALK-positive lung cancer as well as against ALK-positive ALCL cells in vitro (Bonvini et al. 2002; Chen et al. 2010; Katayama et al. 2011; Li et al. 2012; Sequist et al. 2010). Therefore, we determined the efficacy of the potent HSP90 inhibitor AUY922 in Karpas299CR and Karpas299CHR cells. AUY922 potently suppressed growth of Karpas299 parental cells as well as both resistant cell lines, albeit it had slightly weaker effect on Karpas299CHR cells, particularly at a dose of 41 nM (*p* ≤ 0.001) (Fig. 3a). Moreover, observed effect of growth inhibition was dose independent. Similar results were obtained in proliferation assay where BrdU incorporation was measured by flow cytometry. As shown in Fig. 3b, AUY922 treatment at 100 nM resulted in almost complete inhibition of Karpas299 parental cell proliferation as assessed by the number of cells in S phase (Supplementary Fig. S1d). Comparable effect was observed in the case of Karpas299CR and Karpas299CHR cells. Consistent with the cell viability and proliferation data, AUY922 treatment decreased both pALK and total ALK levels with similar potencies in the parental and both resistant cell lines. Also the phosphorylation of ALK-dependent downstream signaling proteins like ERK1/2 and STAT3 was diminished to comparable extent in resistant and wild-type Karpas299 cells (Fig. 3c). Moreover, we observed dose-dependent increase in the level of HSP90 and STAT3 in all three analyzed cell lines (Fig. 3c).

### mTOR inhibitor everolimus suppresses parental and resistant Karpas299 cells’ growth

**Fig. 4:**
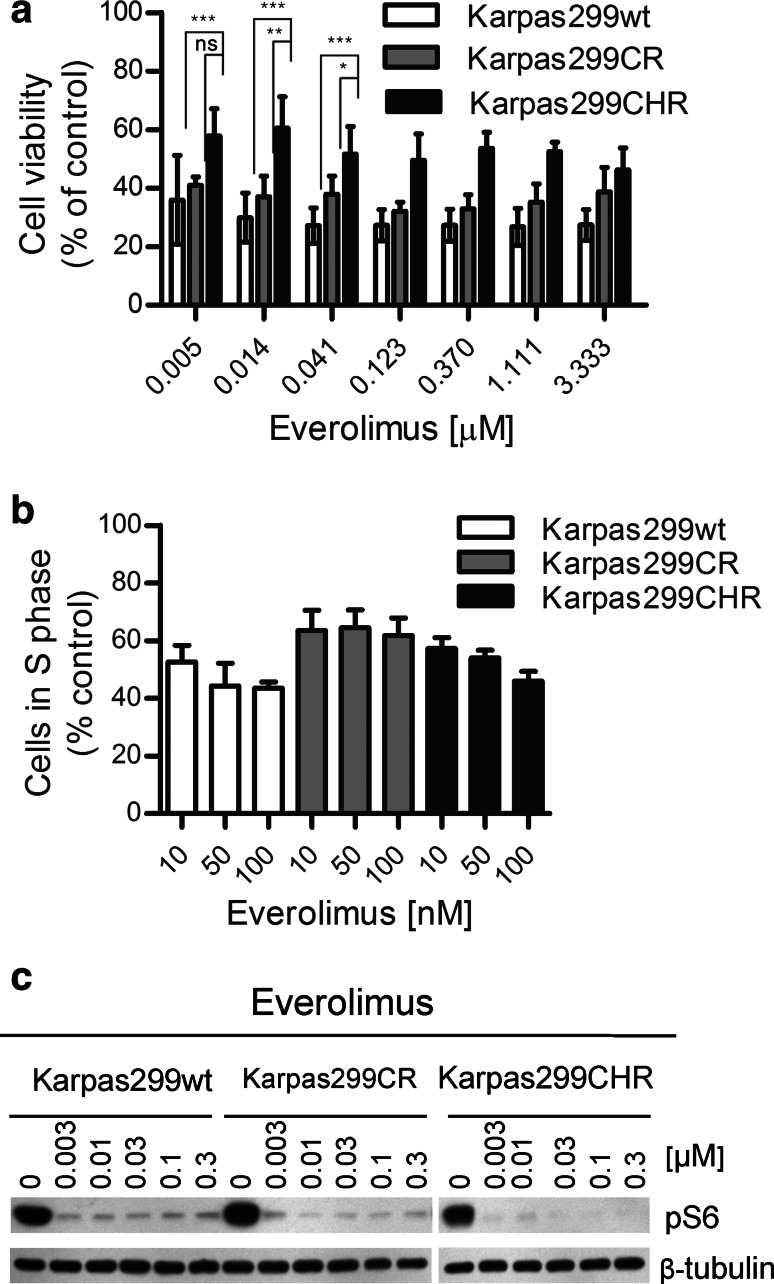
Karpas299CR and Karpas299CHR sensitivity to mTOR inhibitor everolimus. **a** ATPlite cell viability assay following 72-h incubation with everolimus. Values are expressed as percentage viability relative to the untreated controls. Each data point shows the mean of 3 independent triplicate measurements; *error bars* indicate SD. *ns* *p* > 0.05, *p ≤ 0.05, **p ≤ 0.01, ***p ≤ 0.001 (Tukey’s multiple comparison test) for the indicated comparisons. **b** BrdU cell proliferation assay and flow cytometry analysis following 48-h incubation with everolimus. Data expressed as percentage of cells in S phase relative to the untreated controls. Values represent the mean ± SD of two 2 independent experiments. **c** Immunoblot analysis of pS6 following 2-h incubation with everolimus

To identify additional alternative therapy which potentially might be applied for treatment of ALCL patients resistant to ALK inhibitors, we tested the efficacy of different small-molecule inhibitors on growth inhibition of resistant and parental Karpas299 cells (data not shown). We found that everolimus, an inhibitor of mammalian target of rapamycin (mTOR), suppressed growth of parental and resistant cells at low nanomolar concentrations, albeit it demonstrated a little weaker efficacy against Karpas299CHR cells (*p* < 0.0001) (Fig. 4a). Furthermore, everolimus reduced the percentage of proliferating cells of both resistant cell lines at low nanomolar concentrations (Fig. 4b, Supplementary Fig. S1e). However, these effects were slightly weaker in the case of resistant as compared with parental Karpas299 cells. Notably, all tested cell lines responded to everolimus in a dose-independent manner. Moreover, incubation with everolimus did not completely inhibit proliferation of parental and resistant Karpas299 cells up to 100 nM. Immunoblot analysis showed that everolimus decreased phosphorylation of S6 ribosomal protein (S6), one of the main downstream effectors of mTOR, with similar potency in the parental and both resistant cell lines (Fig. 4c). However, we did not observe any impact on phosphorylation status of ALK and its downstream effectors such as ERK1/2 and STAT3 (Supplementary Fig. S2).

## Discussion

Crizotinib demonstrates impressive initial antitumor activity in ALK-positive cancers. However, frequently observed development of drug resistance causes disease progression and limits further clinical benefit of crizotinib-based therapy (Choi et al. 2010; Doebele et al. 2012; Katayama et al. 2011, 2012). In this study, to predict the mechanisms of resistance, which can possibly develop in ALCL patients during crizotinib treatment, we managed to obtain human NPM-ALK+ ALCL Karpas299 cells able to survive and proliferate at high crizotinib concentrations. Additionally, we established Karpas299 cells resistant to the second-generation ALK inhibitor, CH5424802, which is proposed as a second-line treatment in patients who failed to respond to crizotinib (Sakamoto et al. 2011). At the time of preparing this manuscript, there was no data on possible mechanisms of resistance to ALK inhibitors in ALK+ model of ALCL. However, most recently, Ceccon et al. (2013) have established two human NPM-ALK+ ALCL cell lines, KARPAS299 and SUP-M2, resistant to crizotinib. They discovered that a single mutation conferred resistance in each cell line, namely L1196Q and I1171N in Karpas299 and SUP-M2 cells, respectively (Ceccon et al. 2013). Similarly, we discovered that the mechanisms of resistance to crizotinib and CH5424802 in our Karpas299-based models resulted from secondary mutations in ALK KD. We identified I1171T mutation in Karpas299CR, whereas in Karpas299CHR a novel F1174C mutation is not reported in ALCL so far. In our Karpas299CR model, the I1171T mutation became predominant in crizotinib concentration above 1 μM and was present at as high concentration as 2 μM (data not show). In the case of Karpas299CHR cells, we could not exceed CH5424802 concentration of 100 nM. It is probable that F1174C mutation, which emerged in Karpas299CHR cells, is sufficient to confer resistance at 100 nM, but not at higher concentrations of CH5424802.

Mutations of residues F1174 and I1171 were previously reported in neuroblastoma and crizotinib-resistant ALK+ cell lines and patients (Ceccon et al. 2013; Chen et al. 2008; George et al. 2008; Janoueix-Lerosey et al. 2008; Mosse et al. 2008; Sasaki et al. 2010; Zhang et al. 2011). F1174 and I1171 amino acids are not in direct contact with the ATP-binding pocket, where both crizotinib and CH5424802 bind, but they are located in the vicinity of the kinase DFG motif of the activation loop. F1174 and I1171 are important in the regulation of active/inactive conformation of ALK KD since I1171 is a part of hydrophobic spine and F1174 is the central residue of a hydrophobic cluster (Bossi et al. 2010; Kornev et al. 2006). Therefore, the resistance caused by these mutations is not a result of steric hindrance for inhibitor binding, but it rather promotes the active conformation of ALK KD (Sasaki et al. 2010; Zhang et al. 2011). Consistent with this, we observed a substantially higher ALK phosphorylation in Karpas299 cells carrying I1171T and F1174C mutations.

I1171T and F1174C mutations made Karpas299 cells significantly less sensitive to inhibition with ALK TKIs. Karpas299CR and Karpas299CHR cells showed diminished response to all tested ALK inhibitors compared to wild-type Karpas299 cells. In the case of both crizotinib and CH5424802, we observed similar fold increase in IC_50_ values against both resistant cell lines, whereas TAE684 showed lesser increase in IC_50_ value against Karpas299CR and substantially higher against Karpas299CHR. This may suggest that I1171T mutation is less susceptible to TAE684 compared to F1174C. Although I1171T and F1174C mutations increased the IC_50_ for CH5424802 and TAE684 against Karpas299 cells, both the IC_50_s were still nanomolar and were substantially below the concentrations of crizotinib required to inhibit growth, proliferation and ALK phosphorylation in the Karpas299CR and Karpas299CHR cells. These data suggest that more potent and selective ALK inhibitors such as CH5424802, which allow to increase a treatment dose without reaching a dose-limiting toxicity (DLT), can still be effective in ALK+ patients with acquired resistance due to an ALK KD mutation (Seto et al. 2013).

One of the strategies to deal with TKI cancer resistance is administration of an alternative therapy. Promising results have been shown with HSP90 inhibitors (Bonvini et al. 2002; Chen et al. 2010; Katayama et al. 2011; Li et al. 2012; Sequist et al. 2010), like AUY922 which is currently tested in ALK+ NSCLC patients as single agent or in combination with ALK inhibitor LDK378 (NCT01124864, NCT01752400, NCT01772797). ALK fusion proteins are HSP90 clients and require its activity for maturation and maintaining their structural stability (Bonvini et al. 2002; Chen et al. 2010). In our study, AUY922 exhibited similar potencies in the parental and resistant Karpas299 cells, indicating that HSP90 inhibitors might represent an alternative therapeutic strategy for the management of ALK+ ALCL not only in naïve patients but also in patients relapsed due to acquisition of resistance. Similar results have been shown on Ba/F3 lines expressing mutant forms of EML4-ALK and in crizotinib-resistant NSCLC cell line H3122 (Katayama et al. 2011, 2012; Sasaki et al. 2010). Intriguingly, we observed dose-dependent increase in the level of STAT3 in all three analyzed cell lines after treatment with AUY922 (Fig. 3c). As far as observed, increase in the HSP90 level is a normal phenomenon following HSP90 inhibitor treatment and it is an indicator of stress response to HSP90 inhibition, the reason for STAT3 level increase is not clear. To the best of our knowledge, an increase in STAT3 level after HSP90 inhibition was not previously reported. However, it has been established that HSP90 inhibitors compete with heat-shock factor 1 (HSF1) for binding to HSP90 causing release of HSF1 from the inhibitory complex and upregulation of transcription of heat-shock response genes (Georgakis et al. 2006; Sharp and Workman 2006; Zou et al. 1998). We hypothesize that similar to activation of HSP90 expression, release of HSF1 might enhance transcription of *STAT3* gene.

Additionally, we observed that mTOR inhibitor everolimus inhibited growth and proliferation of Karpas299 at low nanomolar concentrations. mTOR has been shown to be activated in ALK+ ALCL cell lines and in tumors (Marzec et al. 2007a; Vega et al. 2006). mTOR inhibition with rapamycin or silencing of mTOR coding gene caused cell cycle arrest and apoptosis in ALK+ ALCL cells, suggesting that mTOR inhibitors might be effective in the therapy of ALK-induced malignancies (Marzec et al. 2007a; Vega et al. 2006). Moreover, the effectiveness of everolimus in growth inhibition of human ALCL xenograft tumors (SUDHL-1 and Karpas299) suggested that mTOR inhibitors might be effective in naïve ALK+ ALCL patients (Li et al. 2012). In this study, we showed that everolimus exhibited similar potencies against Karpas299CR and parental Karpas299 cells, while viability of Karpas299CHR cells was less impaired. Its effect on proliferation of wild-type as well as both resistant cells was different than AUY922, as it did not completely reduce the fraction of S phase cells at a 100 nM concentration (Fig. 4b). Contrary to our results, it has been recently shown that the proliferation of Karpas299 cells and the growth of Karpas299 xenografts were completely inhibited by everolimus, whereas in both models, a complete resistance to AUY922 was observed. However, it should be pointed out that the 50 nM AUY922 concentration used in our study, corresponding to the highest used by Li et al. (2012), also had no effect on proliferation of Karpas299 cells following 48-h incubation (Li et al. 2012). Surprisingly, in contrast to previously reported results (Marzec et al. 2007a), we did not observe any changes in a phosphorylation status of ribosomal protein S6 following incubation with ALK inhibitors of Karpas299 cells (Supplementary Fig. S3). However, the results of Marzec et al. (2007a, b) were obtained using WHI-P154, a multitarget kinase inhibitor; therefore, the observed decrease in the level of pS6 could result not from specific inhibition of ALK but from inhibition of other kinases, such as PI3K (Marzec et al. 2007a). Taken together, our results provide experimental evidence that mTOR inhibitors might not only be an effective therapy in ALK+ ALCL patients but, what is more important, they could be used as a subsequent clinical treatment after emergence of resistance.

## Conclusions

In conclusion, our results suggest that resistance to crizotinib and the next-generation ALK inhibitor CH5424802 may emerge in ALK+ ALCL patients. I1171T and F1174C mutations caused cross-resistance to all three, structurally unrelated ALK inhibitors tested. However, since the concentration of crizotinib needed to inhibit resistant cells is much higher than concentration corresponding to DLT, it is still possible that more potent and selective ALK inhibitors such as CH5424802 or TAE684 structure-based molecules, with sufficiently wider therapeutic window, could be used as an effective therapy in patients that relapse due to acquired resistance. Finally, our results suggest that HSP90 and mTOR inhibitors can constitute effective alternative therapeutic approach in both naïve and ALK TKI-relapsed ALK+ ALCL patients.

## Electronic supplementary material

Below is the link to the electronic supplementary material.

Supplementary material 1 (DOCX 20 kb)

Supplementary material 2 (TIFF 5617 kb)

Supplementary material 3 (TIFF 832 kb)

Supplementary material 4 (TIFF 979 kb)
