# Supplementary material for: Activating mutations in ALK kinase domain confer resistance to structurally unrelated ALK inhibitors in NPM-ALK-positive anaplastic large-cell lymphoma
Source: J Cancer Res Clin Oncol. 2014 Feb 8;140(4):589–98. doi: 10.1007/s00432-014-1589-3 (PMC3949014; doi:10.1007/s00432-014-1589-3)
Supplement: Supplementary file 1 — Supplementary material 1 (DOCX 20 kb) [file 432_2014_1589_MOESM1_ESM.docx]

**Supplementary Materials**

**submission to Journal of Cancer Research and Clinical Oncology**

Activating Mutations in ALK Kinase Domain Confer Resistance to Structurally Unrelated ALK Inhibitors in NPM-ALK-Positive Anaplastic Large Cell Lymphoma

Daria Zdzalik^1^, Barbara Dymek^1^, Paulina Grygielewicz^1^, Pawel Gunerka^1^, Anna Bujak^1,^, Monika Lamparska-Przybysz^1^, Maciej Wieczorek^1^, Karolina Dzwonek^1,2,*^

^1^Innovative Drugs R&D Department, Celon Pharma Inc., Mokra 41a, 05-092 Lomianki/Kielpin, Poland

^2^Department of Immunology, Center for Biostructure Research, Medical University of Warsaw, Banacha 1a, F Building, 02-097 Warsaw, Poland

***To whom correspondence should be addressed:**

Karolina Dzwonek, Department of Immunology, Center for Biostructure, Medical University of Warsaw, Banacha 1a, F Building, 02-097 Warsaw, Poland

Tel.: +48225992199 Fax: +48225992194 Email: [karolina.dzwonek@gmail.com](mailto:karolina.dzwonek@gmail.com)

**Supplementary figures legends**

**Supplementary Fig. S1** Cell cycle distribution and BrdU incorporation in Karpas299CR, Karpas299CHR and parental cell lines following incubation with Crizotinib (**a)** CH5424802 (**b**) TAE684 (**c**) AUY922 (**d**) and Everolimus (**e**). Cells were incubated with indicated concentrations of inhibitors for 48 hours and cell proliferation was determined by staining with BrdU followed by two-color flow cytometry. The percentage of cells in S phase is shown in frame. Representative graph from one of two separate experiments is shown.

**Supplementary Fig. S2** The phosphorylation status of ALK and its downstream signaling proteins following Everolimus incubation of Karpas299CR, Karpas299CHR and parental Karpas299 cells. Cells were incubated with the indicated concentrations of Everolimus for 2 hours, lysed, and subjected to immunoblot to detect the indicated proteins.

**Supplementary Fig. S3** The phosphorylation status of ribosomal protein S6 following incubation of Karpas299CR, Karpas299CHR and parental Karpas299 cells witch ALK inhibitors. Cells were incubated with the indicated concentrations of Crizotinib and CH5424802 for 2 hours, lysed, and subjected to immunoblot to detect the pS6. β-tubulin was used as loading control.
