# Supplementary figures and images for: Activating mutations in ALK kinase domain confer resistance to structurally unrelated ALK inhibitors in NPM-ALK-positive anaplastic large-cell lymphoma
Source: J Cancer Res Clin Oncol. 2014 Feb 8;140(4):589–98. doi: 10.1007/s00432-014-1589-3 (PMC3949014; doi:10.1007/s00432-014-1589-3)

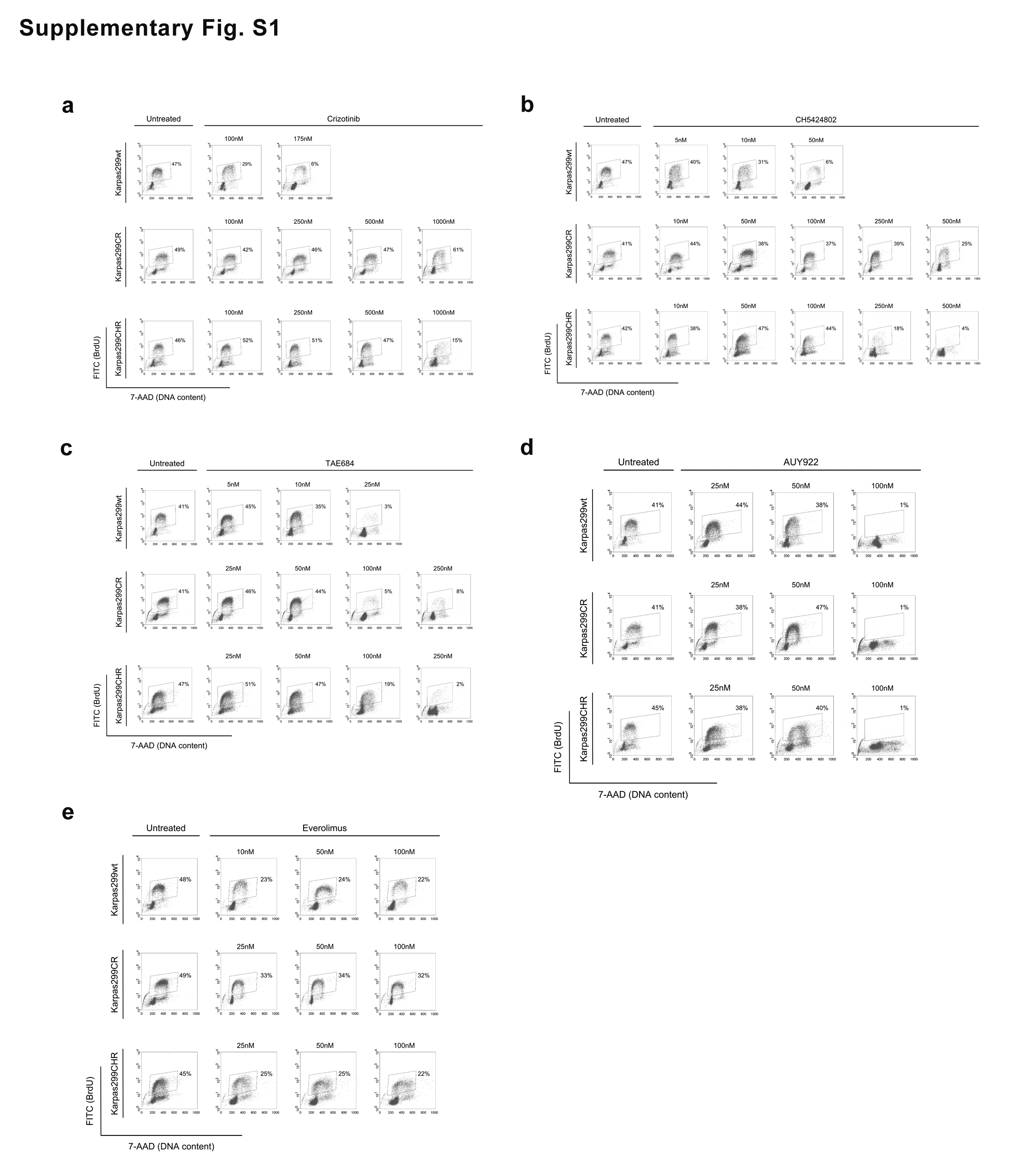

Supplement: Supplementary file 2 — Supplementary material 2 (TIFF 5617 kb) [file 432_2014_1589_MOESM2_ESM.tif]

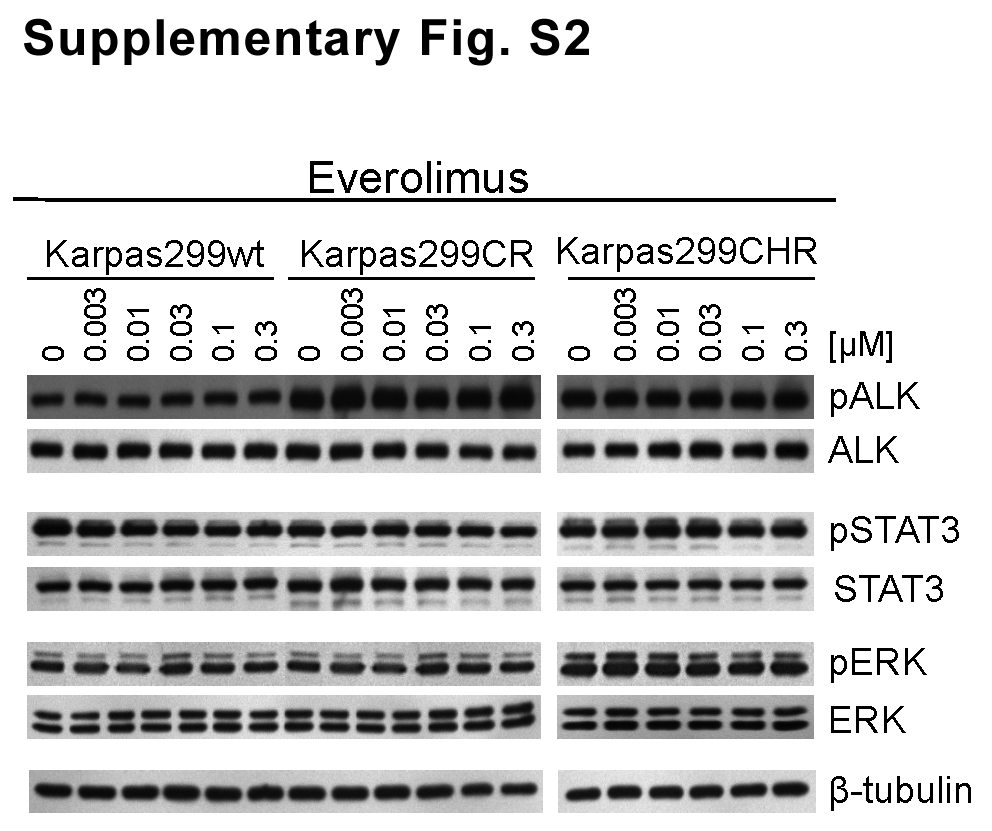

Supplement: Supplementary file 3 — Supplementary material 3 (TIFF 832 kb) [file 432_2014_1589_MOESM3_ESM.tif]

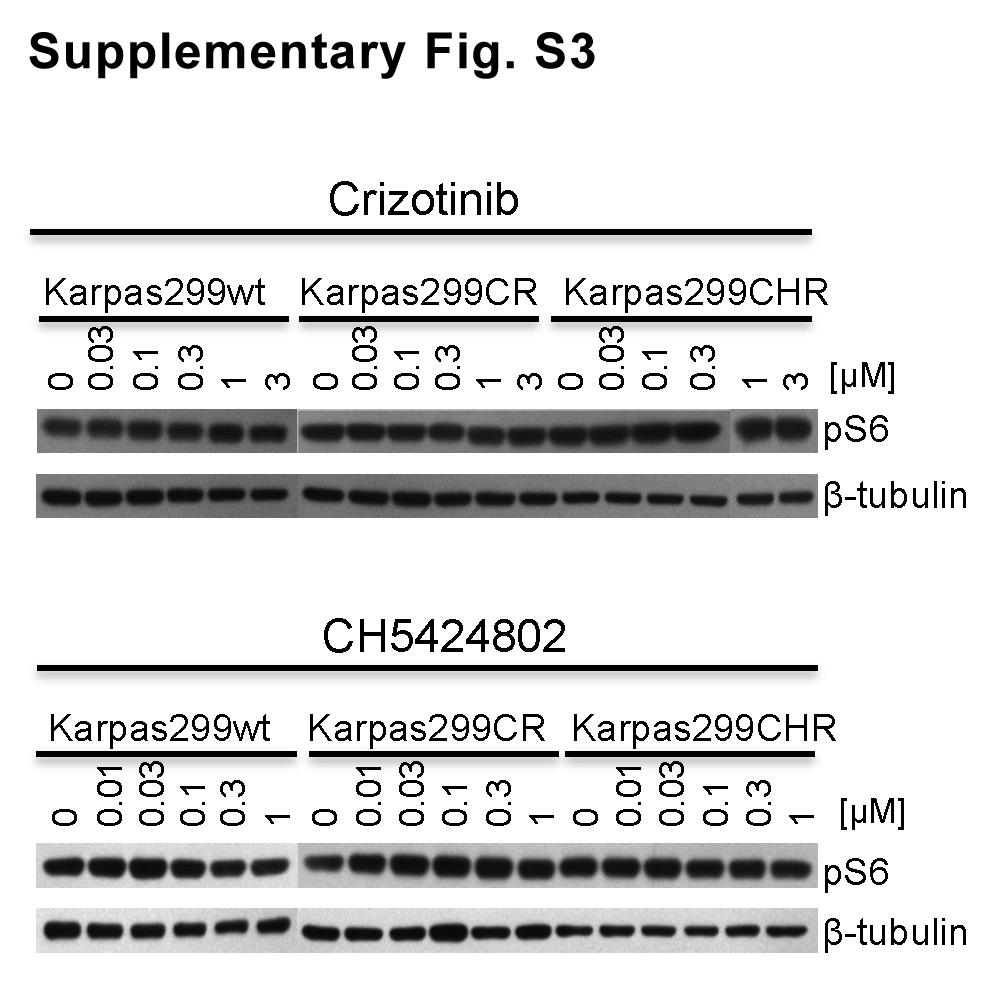

Supplement: Supplementary file 4 — Supplementary material 4 (TIFF 979 kb) [file 432_2014_1589_MOESM4_ESM.tif]
